# The Catalytic Subunit of Protein Phosphatase 1 Gamma Regulates Thrombin-Induced Murine Platelet α_IIb_β_3_ Function

**DOI:** 10.1371/journal.pone.0008304

**Published:** 2009-12-15

**Authors:** Francisca C. Gushiken, Han Hyojeong, Subhashree Pradhan, Kimberly W. Langlois, Nawaf Alrehani, Miguel A. Cruz, Rolando E. Rumbaut, K. Vinod Vijayan

**Affiliations:** 1 Department of Medicine, Baylor College of Medicine, Houston, Texas, United States of America; 2 Department of Pediatrics, Baylor College of Medicine, Houston, Texas, United States of America; 3 Medical Care Line, Michael E DeBakey VA Medical Center, Houston, Texas, United States of America; University of Oldenburg, Germany

## Abstract

**Background:**

Hemostasis and thrombosis are regulated by agonist-induced activation of platelet integrin α_IIb_β_3_. Integrin activation, in turn is mediated by cellular signaling via protein kinases and protein phosphatases. Although the catalytic subunit of protein phosphatase 1 (PP1c) interacts with α_IIb_β_3_, the role of PP1c in platelet reactivity is unclear.

**Methodology/Principal Findings:**

Using γ isoform of PP1c deficient mice (PP1cγ^−/−^), we show that the platelets have moderately decreased soluble fibrinogen binding and aggregation to low concentrations of thrombin or protease-activated receptor 4 (PAR4)-activating peptide but not to adenosine diphosphate (ADP), collagen or collagen-related peptide (CRP). Thrombin-stimulated PP1cγ^−/−^ platelets showed decreased α_IIb_β_3_ activation despite comparable levels of α_IIb_β_3_, PAR3, PAR4 expression and normal granule secretion. Functions regulated by outside-in integrin α_IIb_β_3_ signaling like adhesion to immobilized fibrinogen and clot retraction were not altered in PP1cγ^−/−^ platelets. Thrombus formation induced by a light/dye injury in the cremaster muscle venules was significantly delayed in PP1cγ^−/−^ mice. Phosphorylation of glycogen synthase kinase (GSK3)β-serine 9 that promotes platelet function, was reduced in thrombin-stimulated PP1cγ^−/−^ platelets by an AKT independent mechanism. Inhibition of GSK3β partially abolished the difference in fibrinogen binding between thrombin-stimulated wild type and PP1cγ^−/−^ platelets.

**Conclusions/Significance:**

These studies illustrate a role for PP1cγ in maintaining GSK3β-serine9 phosphorylation downstream of thrombin signaling and promoting thrombus formation via fibrinogen binding and platelet aggregation.

## Introduction

Agonist-induced platelet activation is critical for maintaining hemostasis at the site of vascular injury. Activation of platelets also contributes to the process of thrombosis following the rupture of atherosclerotic plaques. Thrombin generated at the site of injury binds to protease activated receptors (PAR)1 or PAR4 on human or mouse platelets, triggers protein phosphorylation on serine/threonine (Ser/Thr) residues and facilitates integrin α_IIb_β_3_ activation [1]. In particular, Ser/Thr kinases such as mitogen-activated protein kinase (MAPK) [2], protein kinase G (PKG) [3], [4], protein kinase B/AKT [5], [6], protein kinase C α (PKC α) [7], PKC θ [8] and glycogen synthase kinase 3β (GSK3β) [9] play a key role in α_IIb_β_3_ activation by thrombin or PAR4 activating peptide (PAR4-AP). Importantly, thrombin-induced and Ser/Thr kinase–mediated activation of signaling circuitry in platelets is reversible. Since protein phosphatases can fine-tune kinase-mediated signaling processes, we hypothesized that Ser/Thr phosphatases participate in hemostasis/thrombosis by regulating agonist-induced platelet activation.

Platelets express several Ser/Thr phosphatases, and a pool of the catalytic subunits of protein phosphatase 1 (PP1c) and protein phosphatase 2A (PP2Ac) associates with integrin α_IIb_β_3_ [10], [11]. Generic Ser/Thr phosphatase inhibitors like okadaic acid and calyculin A inhibited agonist-induced platelet aggregation, secretion [12]–[14], adhesion and spreading to immobilized fibrinogen [15]. Since these pharmacological agents inhibit several closely related phosphatases (namely, PP1, PP2A and PP4) [16], it is difficult to interpret the specific contribution of protein phosphatase 1 (PP1) and/or its isoforms in agonist-induced integrin α_IIb_β_3_ signaling. Our goal was to address a role for PP1 in platelets using a genetic approach.

PP1 is a major eukaryotic Ser/Thr protein phosphatase that regulates a variety of functions like glycogen metabolism, muscle contraction, transcription, translation and cell division [17], [18]. PP1 is a multimeric enzyme formed by the assembly of catalytic and regulatory subunits. PP1c is a 35–38 kDa protein that exists as three isoforms: α, β/δ and γ sharing greater than 90% amino acid sequence similarity. Two splice variants of PP1cγ (PP1cγ1 and PP1cγ2) have also been identified. All PP1c isoforms are ubiquitously expressed, except for PP1cγ2 that is testis specific. Cellular PP1 activity is regulated by multiple factors: 1) reversible phosphorylation of the regulatory subunits 2) dissociation of the regulatory and the catalytic subunits 3) allosteric regulation of the regulatory subunits and 4) inducible expression of the regulatory subunits [17].

Among the various isoforms of PP1c, mice bearing a targeted deletion of the gene for PP1cγ [*Ppp1cc* ^−/−^] are viable and are considered in this study [19]. Using PP1cγ^−/−^ platelets, we report a moderate decrease in low dose thrombin-induced α_IIb_β_3_ activation, soluble fibrinogen binding, platelet aggregation and thrombus formation. GSK3β-Ser9 phosphorylation that promotes platelet function was also decreased in thrombin-stimulated PP1cγ^−/−^ platelets. GSK3β inhibitor partially abrogated the difference in fibrinogen binding between thrombin-stimulated wild type and PP1cγ-/- platelets. These studies suggest a positive role for PP1cγ in thrombin-induced platelet functional responses.

## Materials and Methods

### Materials

Unless stated, all reagents were from Sigma-Aldrich (St. Louis, MO). Fluorescein isothiocyanate (FITC)-conjugated antibodies (anti-mouse CD41 (α_IIb_) and CD62 (P-selectin) were from BD Bioscience (San Jose, CA). Phycoerythin-conjugated JON/A (recognizes activated murine α_IIb_β_3_) and thrombin were gifts from Dr. B. Nieswandt (University of Wurzburg, Germany) and Dr. J. Fenton (New York State Department of Health, Albany), respectively. ADP and collagen were from Helena Laboratories (Beaumont, TX). GSK3β inhibitor VIII was from Calbiochem EMD4Biosciences (Darmstadt, Germany). Protease-activated receptor 4-activating peptide (PAR4AP) AYPGKF was synthesized by the Protein Core Facility at Baylor College of Medicine. Collagen-related peptide (CRP) was synthesized at Baylor College of Medicine and cross-linked by glutaraldehyde. Alexa 488-conjugated fibrinogen was from Invitrogen, (Carlsbad, CA) while human fibrinogen was from Enzyme Research Laboratories Inc. (South Bend, IN). Antibodies to phospho-AKT Ser473, phospho-GSK3β Ser9 and AKT were obtained from Cell signaling (Boston, MA). Antibodies to theα, and γ isoforms of PP1c, PAR3 and PAR4 were purchased from Santacruz Biotechnology (Santacruz, CA). β isoform of PP1c was from Upstate Biotechnology/Millipore (Billerica, MA).

### Mice Platelet Preparation

All animal studies were approved by the Institutional Animal Care and Use Committee at Baylor College of Medicine. CD-1 mice carrying the PP1cγ targeted deletion [19] and subsequently backcrossed for 10 generations onto Balb/C background were obtained from Dr. S. Varmuza (University of Toronto). For most studies, 8–14 weeks old wild type (WT) and PP1cγ^−/−^ littermate mice matched for age and gender were used. Blood was collected from the inferior vena cava of isoflurane-anesthetized mice into acid-citric acid-dextrose (ACD) at a ratio of 1∶10 (vol/vol). Whole blood was further diluted with an equal volume of Dulbecco's phosphate buffered saline (D-PBS) (Invitrogen), [2.6 mM KCl, 1.4 mM KH_2_PO_4_, 137 mM NaCl, 8 mM Na_2_HPO_4_] containing ACD (1∶9 parts ACD/D-PBS). Platelet rich plasma (PRP) was obtained by centrifugation of the diluted blood at 68 g for 10 minutes at room temperature. PRP was further centrifuged at 754 g for10 minutes and the resulting platelet pellet was washed once in D-PBS and resuspended in D-PBS containing 0.005 U/ml apyrase to avoid desensitization of ADP receptors. Platelets were counted using a Coulter counter (Beckman-Coulter (Z1), Miami, FL) and adjusted to a final concentration of 2.5×10^8^/ml and then allowed to rest for at least 1 hour.

### Immunoblotting Studies

Resting platelets were lysed with 1% Triton X-100 containing lysis buffer supplemented with a cocktail of protease and phosphatase inhibitors. Forty µg of protein lysate were resolved on 10% sodium dodecyl sulfate-polyacrylamide gel electrophoresis (SDS-PAGE) and immunoblotted with antibodies to different isoforms of PP1c, PAR3, PAR4 and actin. In some experiments, washed platelet treated with varying concentrations of thrombin for 2 minutes were lysed and immunoblotted with antibodies to phospho-AKT Ser 473, phospho-GSK3β Ser9, and AKT. Binding of primary antibodies were detected by HRP-conjugated secondary antibodies developed using ECL-system (Amersham Biosciences).

### Fibrinogen Binding, Flow Cytometry Analysis and Platelet Aggregation

To study fibrinogen binding, washed platelets were diluted to a concentration of 2.5×10^7^/ml with Tyrode's buffer containing 1.8 mM CaCl_2_ and 0.49 mM MgCl_2_. Platelets were stimulated with various concentrations of the following agonists: thrombin (0.005, 0.01, 0.02, 0.1 U/ml), adenosine diphosphate (ADP) (5, 10, 20 µM), collagen (1, 2.5 and 5 µg/ml), collagen-related peptide (CRP) (0.1, 0.2 µg/ml), PAR4-AP (100, 500 µM) followed by addition of Alexa 488-conjugated fibrinogen (50 µg/ml final concentration) at room temperature for 15–20 minutes. In some studies, platelets were pretreated with control (DMSO) or 10 µM GSK3β inhibitor VIII for 30 minutes before stimulation with thrombin (0.02 U/ml). In other studies, platelets (resting or thrombin-stimulated) were incubated with FITC-conjugated anti-mouse CD41, PE-conjugated anti-mouse GPIbα, PE-labeled JON/A, and FITC-conjugated anti-mouse P-selectin antibodies to detect α_IIb_β_3_, GPIbα expression, activation of integrin α_IIb_β_3_, and α granule secretion, respectively. Fluorescence-activated cell sorting (FACS) analysis of platelets was performed using an EPICS-XL flow cytometry (Beckman Coulter). Platelet aggregation in response to various agonists was initiated by addition of various agonists to a 225 µl aliquot of washed platelets in a four channel Bio/Data PAP-4C aggregometer (Biodata Corporation, Horsham, PA)

### Platelet Adhesion and Clot Retraction

Ninety six well plates were coated with 100 µg/ml of fibrinogen and blocked with 5 mg/ml of Bovine Serum Albumin (BSA). Control wells were coated with only BSA. Fibrinogen or BSA coated wells were incubated with 1×10^7^ washed platelets in D-PBS supplemented with 1.8 mM CaCl_2_ and 0.49 mM MgCl_2_ for varying time periods at 37°C. Unbound platelets were washed and the attached platelets were quantified by assaying for acid phosphatase activity at 405 nM. The number of platelets attached was obtained by using a standard curve for absorbance *versus* cell number. Fibrin-clot retraction, was initiated by the addition of 1 U/ml thrombin to 150 µl of mouse platelet-rich plasma containing (2.5×10^8^ platelets/ml) supplemented with 3 mM CaCl_2_. After incubating at RT for varying time periods, the amount of liquid not incorporated into the clot is measured. The volume of the clot was determined by subtracting the measured volume from 150 µl (initial volume). Clot volume was expressed as a percentage of the initial volume.

### In Vivo Platelet Thrombus Formation

Microvascular thrombosis was examined in the cremaster venules using a previously well described model of light/dye –induced endothelial injury by intravital microscopy [20], [21]. Briefly, male mice anesthetized with 50 mg/kg phenobarbital sodium were placed on a custom plexiglas tray maintained at 37°C with a homeothermic blanket. To assist in breathing, a tracheotomy was performed. Later, an internal jugular vein and common carotid artery were cannulated to facilitate administration of various preparations and monitoring of blood pressure/heart rate, respectively. The cremaster microvascular bed was prepared, perfused with bicarbonate-buffered saline solution (pH range, 7.35–7.45) at 35°C and transferred to the stage of an upright intravital video microscope (BX-50; Olympus, Tokyo, Japan). After equilibration for 30 minutes, 10 mL/kg 5% FITC-labeled dextran (150 kD) was injected intravenously through the jugular vein. A suitable venule was selected after survey of the vascular bed with a 4× objective (NA 0.13) lens. Following the measurement of the diameter and blood flow velocity of the vessel (Doppler velocimeter; Microcirculation Research Institute, College Station, TX), a photochemical injury was initiated by exposing approximately 100 µm of the vessel to filtered excitation light at 0.6 W/cm^2^ (from a 175W xenon lamp; Sutter Instrument, Novato, CA; and an HQ-FITC filter cube; Chroma Technology, Brattleboro, VT). Epi-illumination was applied continuously, and the time of onset of platelet aggregates (thrombus onset) and the time of flow cessation (for at least 60 seconds) were monitored and recorded using a 40× water-immersion objective (NA 0.8) by an observer who was blinded to the genotype of the animals. Thrombi were induced in 1–2 venules per animal, and the results for each animal were averaged.

### Tail-Bleeding Times

Tails of anesthetized mice (WT and PP1cγ^−/−^) were severed with a scalpel 1 mm from the tip and immediately immersed in a tube containing PBS solution at 37°C. Thirty seconds later, the tail was immersed into a new tube with PBS and this process of transferring the tail to a new tube was repeated until the bleeding stopped. The time from the incision to cessation of blood (as revealed by visual examination of blood in the tubes) were recorded as tail bleeding times.

### Statistical Analysis

Results are expressed as means +/− SEM. Experimental conditions were compared by using paired student's *t* test.

## Results

### PP1cγ ^−/−^ Platelets Exhibited Comparable Expression of Other PP1c Isoforms

**Figure 1:**
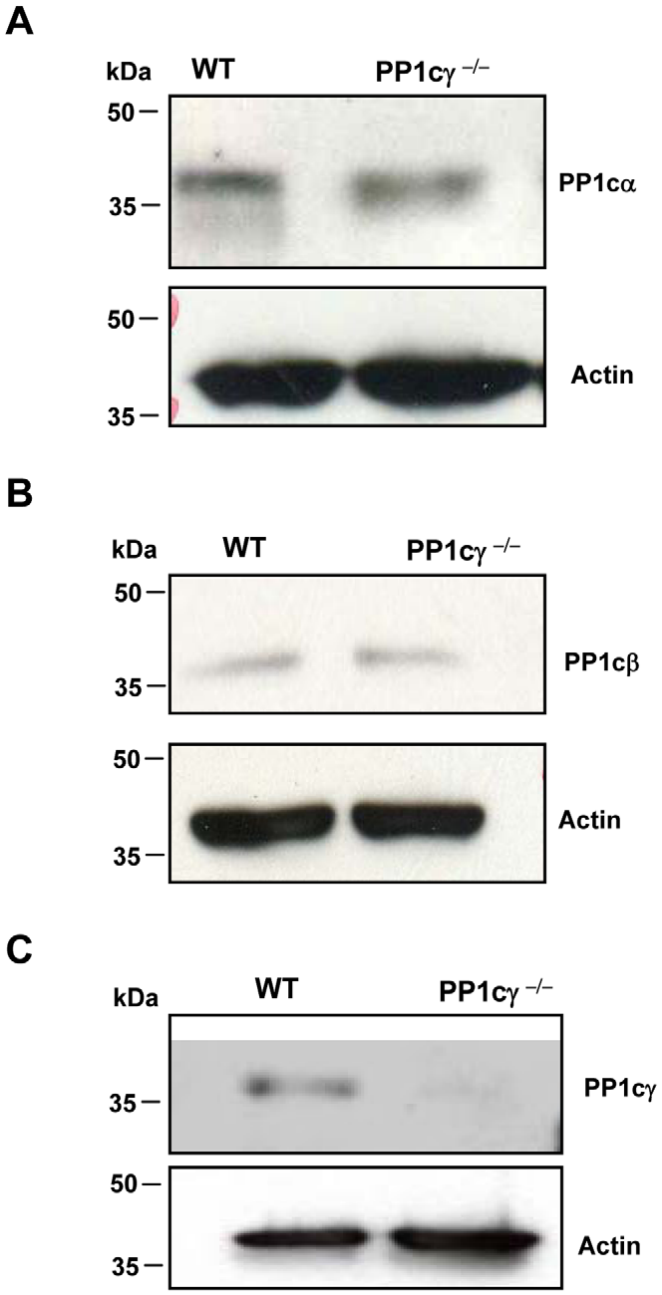
PP1c isoforms in platelets. (**A–C**) Platelet lysate from wild type (WT) or PP1cγ^−/−^ mice were evaluated by SDS-PAGE and Western blotting using isoform specific antibodies to α, β and γ (recognizes γ1 and γ2 splice variants). These membranes were stripped and reprobed with actin to demonstrate equal protein loading (lower panels). Blots are representative of three independent experiments.

Immunoblotting studies with an antibody specific for the γ isoform of PP1c confirmed the absence of PP1cγ protein in PP1cγ^−/−^ platelets (Figure 1C). Furthermore, there was no evidence for a compensatory up-regulation of α and β isoforms of PP1c in the platelets from PP1cγ^−/−^ mice (Figure 1A and 1B). These blots were stripped and reprobed with actin to ensure equal protein loading (Figure 1 A, Band C lower panel).

### PP1cγ ^−/−^ Platelets Exhibited a Mild Agonist-Specific Impairment in Fibrinogen Binding and Aggregation

**Figure 2:**
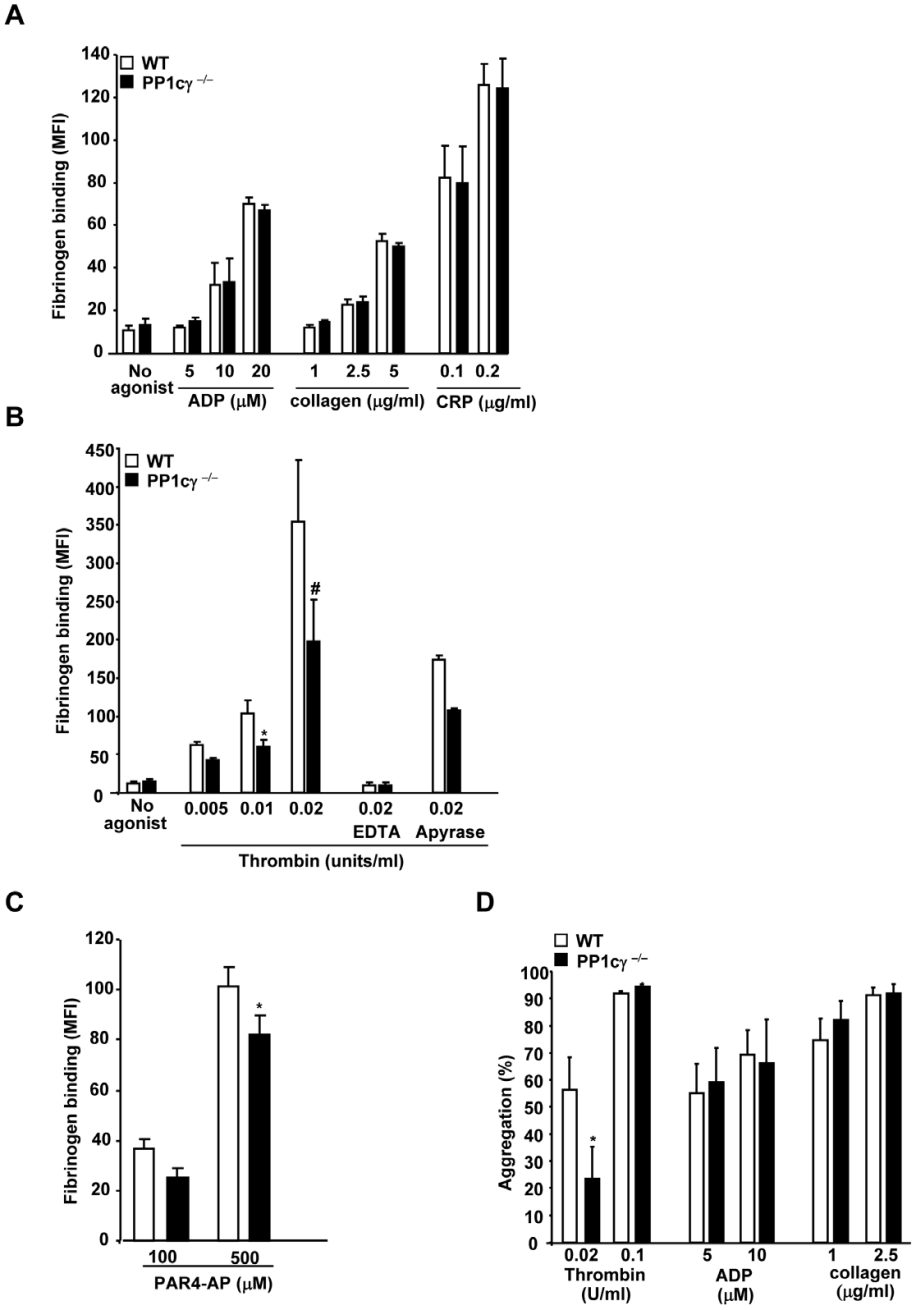
Thrombin specific impairment in soluble fibrinogen binding and aggregation in PP1cγ^−/−^ platelets. Washed platelets from WT or PP1cγ^−/−^ mice were stimulated with ADP, collagen or CRP (**A**), thrombin (**B**) or PAR4-AP (**C**) in the presence of Alexa 488 fibrinogen and bound fibrinogen analyzed by flow cytometry as mean fluorescence intensity (MFI). Data are ±SEM of 9–11 experiments for ADP, thrombin and collagen and 13–14 experiments for PAR4-AP. As compared to the WT platelets, the decreased fibrinogen binding in PP1cγ^−/−^ platelets were significant at *p = 0.02 (for 0.01 U/ml thrombin); #p = 0.01 (for 0.02 U/ml thrombin); *p = 0.05 (for 200 µM PAR4-AP). In other experiments (n = 3), platelets were pretreated with 0.5 mM EDTA or 2 mM apyrase (ADP scavenger) prior to stimulation with 0.02 U/ml thrombin. (D) Percentage aggregation for WT and PP1cγ^−/−^ platelets in response to the indicated doses of thrombin, ADP and collagen. Studies are indicated as ±SEM of 5–6 experiments. The decreased aggregation in 0.02 U/ml thrombin stimulated PP1cγ^−/−^ platelets compared to WT platelets was significant at *p = 0.04.

To determine if the loss of *Ppp1cc* gene affected platelet reactivity washed platelets from the WT and PP1cγ^−/−^ mice were stimulated with platelet agonists and analyzed for soluble fibrinogen binding. Low concentrations of strong agonists like thrombin, collagen and dilute suspension of platelets were used to ensure that platelets did not aggregate in this assay. ADP, collagen and CRP induced-soluble fibrinogen binding were comparable between the WT and PP1cγ^−/−^ platelets (Figure 2A). In contrast, addition of low doses of thrombin to PP1cγ^−/−^ platelets resulted in a modest but significant decrease in the soluble fibrinogen binding. A ∼42% (p = 0.02) and ∼45% (p = 0.01) decreased fibrinogen binding in response to 0.01 U/ml and 0.02 U/ml of thrombin was observed in PP1cγ^−/−^ platelets (Figure 2B). However, the difference in fibrinogen binding between WT and PP1cγ^−/−^ platelets was lost with increasing concentrations of thrombin (not shown). Addition of cation chelator EDTA known to block α_IIb_β_3_ function inhibited fibrinogen binding in WT and PP1cγ^−/−^ platelets to values obtained in unstimulated platelets. Many, but not all responses of thrombin in human platelets could be reproduced by thrombin receptor activating peptide [22]–[24]. To explore whether PP1cγ participated in thrombin signaling downstream of mouse thrombin receptor PAR4, we stimulated platelets with protease-activated receptor 4 activating peptide (PAR-4AP) and assessed fibrinogen binding. Compared with wild type platelets, PP1cγ^−/−^ platelets showed only marginally decreased soluble fibrinogen binding in response to PAR-4AP (P = 0.05) (Figure 2C).

Soluble fibrinogen binding under stirring conditions can lead to platelet aggregation. Next, we determined whether platelet aggregation in response to agonist was altered in PP1cγ^−/−^ platelets. Compared with WT platelets, PP1cγ^−/−^ platelets demonstrated ∼60% decreased aggregation to 0.02 U/ml thrombin. Aggregation in response to ADP and collagen was comparable between the WT and PP1cγ^−/−^ platelets (Figure 2D). Since moderate differences in soluble fibrinogen binding and aggregation between the WT and PP1cγ^−/−^ platelets were noticed only at low thrombin concentrations and not with other agonists, we conducted the rest of the studies in this report with only low dose thrombin.

### PP1cγ ^−/−^ Platelets Exhibited Decreased Thrombin-Induced Activation of α_IIb_β_3_ despite Normal Expression of α_IIb_β_3_, Thrombin Receptors and Normal Granule Secretion

**Figure 3:**
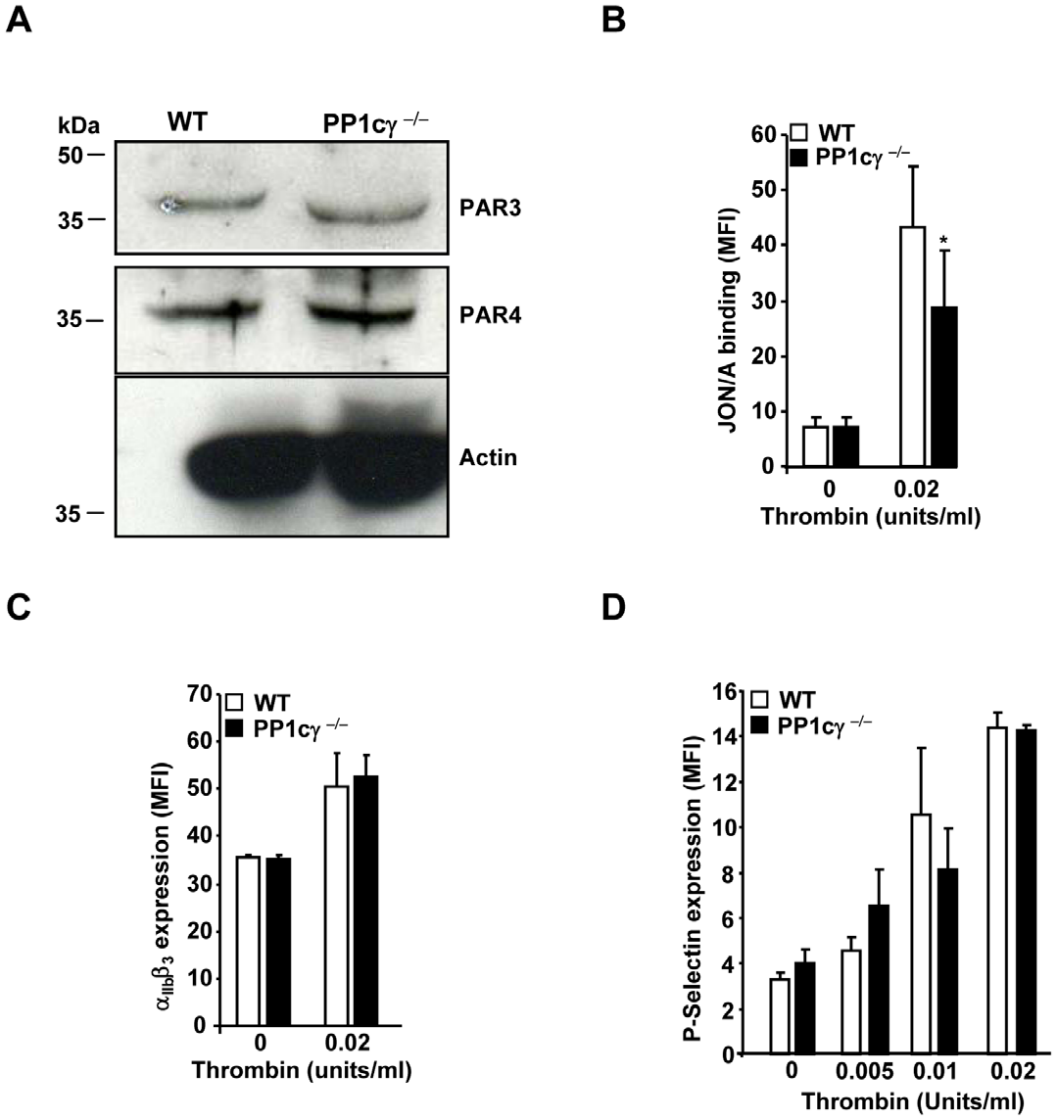
Expression of thrombin receptors, α_IIb_β_3_ and P-selectin in PP1cγ^−/−^ platelets. (**A**) Lysate from resting washed platelets was evaluated by SDS-PAGE and immunoblotted with anti-PAR3 antibody (∼43 kDa). The blot was stripped and reprobed with anti- PAR4 (∼38 kDa) and then with anti-actin (loading) antibodies. Blots are representative of two experiments. Washed platelets were incubated with anti-JON/A PE antibody (**B**) or anti-α_IIb_ FITC antibody (**C**) or anti-P-selectin antibody (**D**) and subjected to flow cytometry. Results are expressed as ±SEM of 5–6 experiments. Compared to thrombin stimulated WT platelets, the decreased binding of JON/A antibody to PP1cγ^−/−^ platelets was significant at *p = 0.003.

To identify potential mechanisms for the decreased reactivity in thrombin stimulated PP1cγ^−/−^ platelets, we analyzed the expression of thrombin receptors PAR3 and PAR4. Because reliable antibodies that detect PAR4 by immunofluoresence were not readily available, we subjected platelet lysate to immunoblotting experiments with anti-PAR3 and PAR4 antibodies. Comparable expression of PAR3 and PAR4 receptors was observed between the WT and PP1cγ ^−/−^ platelets (Figure 3A). Besides PARs, GPIbα serves as high affinity thrombin binding site [25]. However, GPIbα expression was not different in WT and PP1cγ ^−/−^platelets (data not shown). Next, we studied the activation status of integrin α_IIb_β_3_ in thrombin-stimulated WT and PP1cγ^−/−^ platelets by flow cytometry using a murine α_IIb_β_3_ activation–specific JON/A antibody. Under resting conditions, there was no difference in JON/A binding between the WT and PP1cγ ^−/−^ platelets. In contrast, addition of thrombin (0.02 U/ml) to PP1cγ ^−/−^ platelets resulted in a moderate but significant decrease in JON/A binding (Figure 3B). Expression levels of α_IIb_β_3_ in the resting and thrombin stimulated platelets were comparable in the WT and PP1cγ^−/−^ mice (Figure 3C). This indicates that the decreased JON/A binding in thrombin stimulated PP1cγ^−/−^ platelets were not due to the altered integrin expression. Potential alterations in platelet granule secretion events in PP1cγ^−/−^ mice could account for the decreased α_IIb_β_3_ activation at low doses of thrombin. However, secretion of P-selectin (α granule content) was not altered between WT and PP1cγ^−/−^ platelets (Figure 3D). Furthermore, addition of exogenous fibrinogen did not rescue the decreased aggregation of thrombin stimulated PP1cγ ^−/−^ platelets (not shown). ADP scavenger apyrase did not abolish thrombin-induced fibrinogen binding difference between the WT and PP1cγ^−/−^ platelets (Figure 2B). This observation suggests that the fibrinogen binding difference between the WT and PP1cγ ^−/−^ platelets is not dependent on dense granule ADP. Thus, these studies suggest that decreased α_IIb_β_3_ activation and not altered receptor expression or granule secretion may have contributed to the decreased fibrinogen binding in thrombin-treated PP1cγ^−/−^ platelets.

### PP1cγ^−/−^ Platelet Exhibited Normal Outside in Integrin α_IIb_β_3_ Signaling

**Figure 4:**
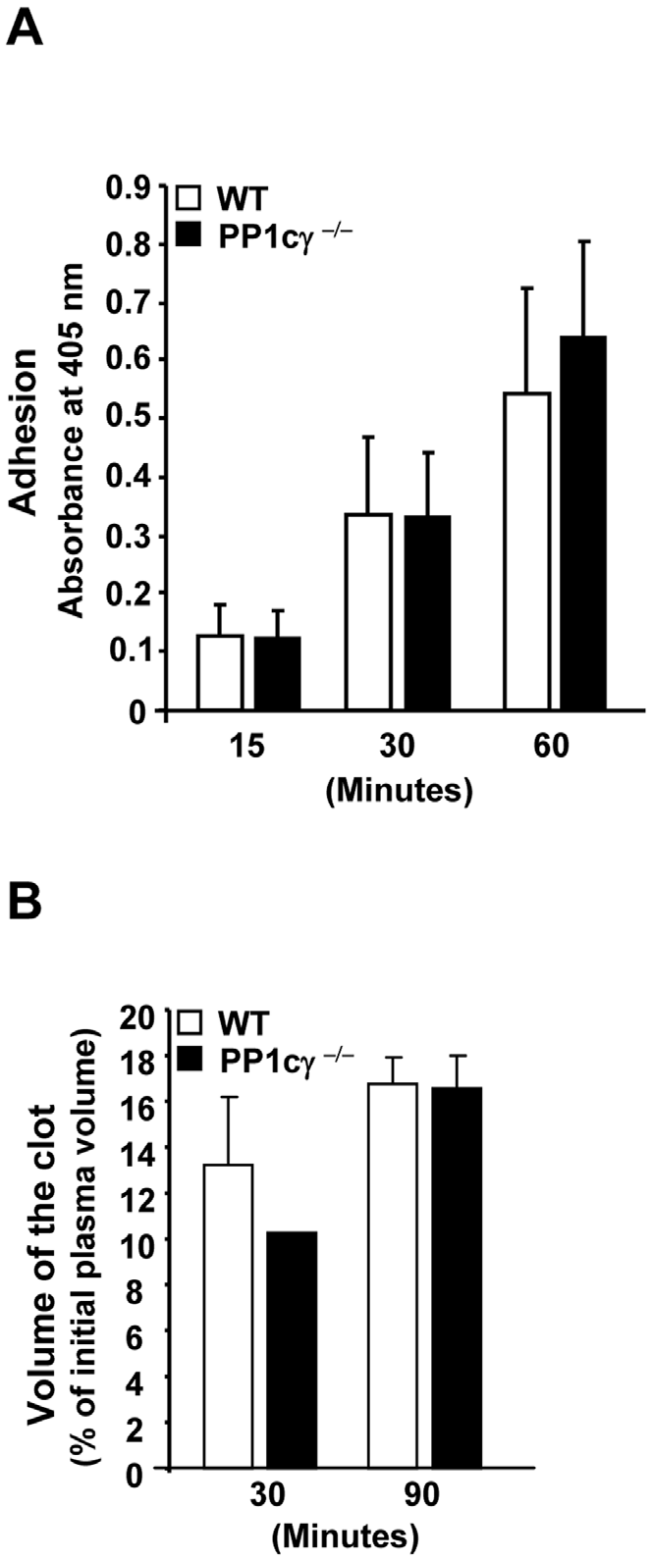
Outside-in integrin α_IIb_β_3_ signaling functions in PP1cγ^−/−^ platelets. (**A**). Washed platelets were incubated with immobilized fibrinogen-coated micro titer wells for the indicated periods of time and adhesion quantified. The data are expressed as ±SEM of 3 experiments. (**B**) Fibrin clot retraction was examined following the addition of thrombin to platelet-rich plasma. Volume of the clot is expressed as ±SEM of 3 experiments.

We assessed if functions mediated by outside-in integrin α_IIb_β_3_ signaling were altered in the PP1cγ^−/−^ mice. In contrast to the data from soluble fibrinogen, adhesion of PP1cγ^−/−^ platelets to immobilized fibrinogen was comparable to the WT platelets (Figure 4A). Thrombin-induced clot retraction of platelet rich plasma samples was also not altered between the WT and PP1cγ^−/−^ mice (Figure 4B). Collectively, these studies imply that outside-in integrin α_IIb_β_3_ signaling processes were not altered by the lack of PP1cγ in platelets.

### PP1cγ^−/−^ Mice Exhibited Delayed Thrombus Formation in a Light-Dye Injury Thrombosis Model

**Figure 5:**
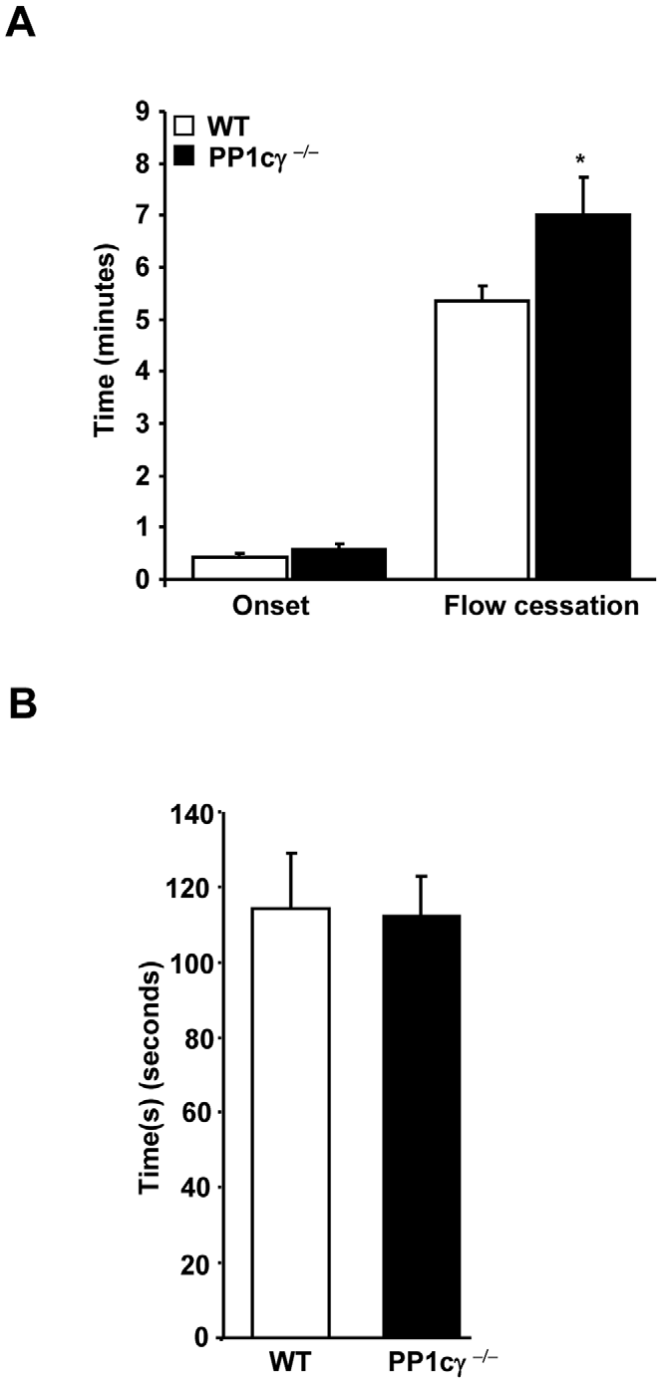
Role of PP1cγ in thrombosis and hemostasis injury models. (**A**). Light/dye -induced microvascular thrombosis was studied in the venules of the cremaster muscles of WT and PP1cγ^−/−^ mice by intra vital microscopy. The onset of thrombosis (onset) and the cessation of blood flow following the injury were monitored. Results are expressed as ±SEM of 13 experiments. Compared to the WT, the delayed time for flow cessation in PP1cγ^−/−^ mice was significant at *p = 0.03. (**B**). Tail vein bleeding times from 13 WT and PP1cγ^−/−^ mice are shown.

To establish if the defect in PP1cγ ^−/−^ platelets observed *in vitro* might affect thrombus formation *in vivo*, we studied the responses of WT and PP1cγ^−/−^ mice to a light/dye-induced injury model that has been previously shown not to denude the endothelium [20]. The initiation time for thrombus formation was not different between the WT and PP1cγ^−/−^ mice (Fig. 5A). In contrast, we noticed a moderate but significant (P = 0.03) delay in the time for complete cessation of blood flow (occlusion time) in PP1cγ^−/−^ mice (Fig. 5A). Microvascular diameter and wall shear rate was comparable between the WT and PP1cγ^−/−^ mice (23.3±0.3 µm vs. 24.6±0.5 µm and 515±28 sec^−1^ vs. 516±29 sec^−1^, respectively). Similarly, blood pressure and heart rate was not significantly different between the WT and PP1cγ^−/−^ mice (82.8±1.9 mmHg vs. 81.5±21. mmHg and 436±14 min^−1^ and 465±12 min^−1^, respectively) suggesting that the delayed thrombus formation in PP1cγ ^−/−^ mice was independent of these above mentioned factors. Thus, in this thrombosis model, PP1cγ participates modestly in stabilizing thrombus formation. In contrast, tail bleeding times were not different between the WT and PP1cγ^−/−^ mice (Figure 5B).

### Thrombin-Stimulated PP1cγ^−/−^ Platelets Exhibited Decreased Glycogen Synthase Kinase3 β -Serine 9 Phosphorylation in an AKT Independent Manner

**Figure 6:**
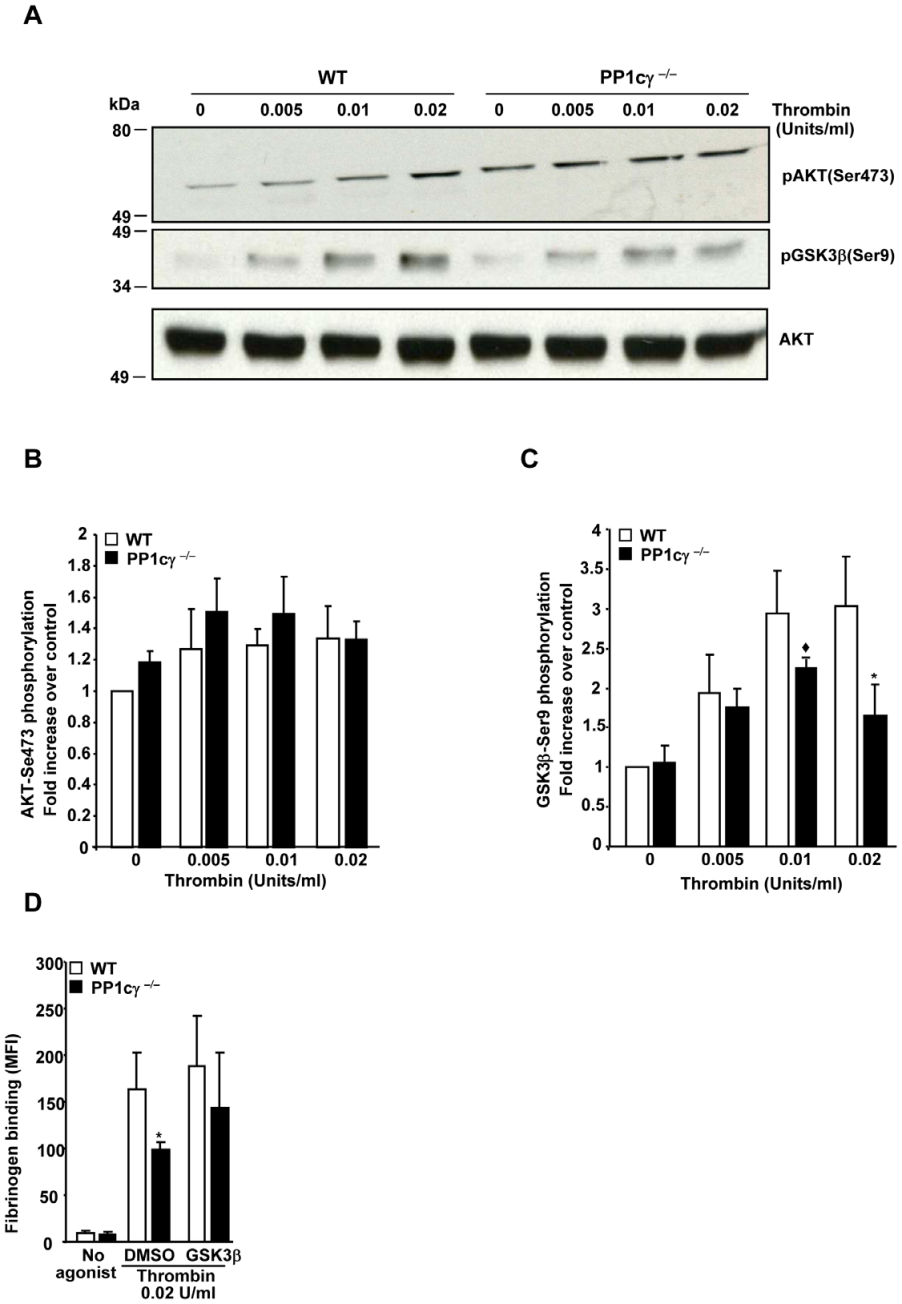
Decreased phosphorylation of GSK3β-Ser9 in thrombin stimulated PP1cγ^−/−^ platelets. (**A**). Platelet lysate from resting (0) and thrombin activated (0.005, 0.01 and 0.02 U/ml) was separated on an SDS-PAGE and immunoblotted with antibodies to AKT-Ser473, GSK3β- Ser9 and AKT (total). (**B and C**) Densitometric quantification of phosphorylation levels for AKT-Ser473 and GSK3β-Ser9 in WT and PP1cγ^−/−^ stimulated platelets. The decreased phosphorylation of GSK3β-Ser9 in thrombin-stimulated PP1cγ^−/−^ platelets was significant at ♦p = 0.05 (for 0.01 U/ml thrombin); *p = 0.02 (for 0.02 U/ml thrombin). Data are expressed as ±SEM of 5 experiments. (**D**) Effect of GSK3β inhibitor VIII on thrombin-induced fibrinogen binding on WT and PP1cγ^−/−^ platelets. Compared with WT platelets, PP1cγ^−/−^ platelets displayed ∼40% decreased fibrinogen binding in response to 0.02 U/ml thrombin in the presence of solvent control (DMSO) (*P = 0.048) and GSK3 inhibitor VIII (GSK3β) partially reduced the difference to 23%. Data are expressed as ±SEM of 3–4 experiments.

Platelet functional responses to thrombin are mediated in part by activation of signaling molecules in the PI3K-AKT-GSK-3β circuitry. Interestingly, there exist some similarly in the phenotype of PP1cγ ^−/−^ mice when compared to AKT2^−/−^, AKT1^−/−^ and GSK3β^+/−^ mice. All four null mice predominantly showed thrombin or PAR4 specific impairment in platelet function except AKT1^−/−^ mice, which also exhibited defects in platelet responses to collagen [6]. Therefore, we investigated if PP1cγ regulates thrombin-induced signaling to AKT. Activation of AKT as measured by phosphorylation of AKT-Ser473 was comparable between the thrombin stimulated WT and PP1cγ ^−/−^ platelets (Figure 6A and 6B). Next, we assessed the activation of GSK3β (AKT substrate) because PP1 can regulate GSK-3β Ser9 phosphorylation, independent of AKT, under in vitro conditions [26]. GSK3β is a suppressor of platelet function and agonist–induced Ser9 phosphorylation decreases GSK3β kinase activity and promotes platelet function [9]. Consistent with the earlier report [9], thrombin stimulation in WT platelets resulted in sustained phosphorylation of GSK-3β-Ser9. Unexpectedly, thrombin-stimulated PP1cγ ^−/−^ platelets showed a maximum of ∼1.9 fold decreased GSK-3β-Ser9 phosphorylation (6A and 6C). These observations imply a role for PP1cγ in GSK-3β-Ser9 phosphorylation downstream of thrombin signaling by an AKT independent pathway. It should be emphasized that although a majority of PAR4-stimulated GSK3β-Ser9 phosphorylation was previously shown to be dependent on PI3K/AKT signaling, the same study also reported a fraction of GSK3β-Ser9 phosphorylation that was independent of PI3K/AKT pathway [9]. Thus, a pathway involving PP1cγ might represent one of the non-AKT dependent mechanisms that could potentially regulate GSK3β-Ser9 phosphorylation.

Since GSK3β-Ser9 phosphorylation inhibits the constitutive GSK3β activity, we examined whether the decreased GSK3β-Ser9 phosphorylation (active GSK3β) upon thrombin stimulation could contribute to the decreased fibrinogen binding in PP1cγ ^−/−^ platelets. Compared with WT platelets, PP1cγ ^−/−^ platelets exhibited ∼40% decreased fibrinogen binding upon stimulation with 0.02 U/ml thrombin in the presence of DMSO (control). However, in the presence of GSK3β inhibitor VIII, PP1cγ ^−/−^ platelets exhibited ∼23% decreased fibrinogen binding compared to the WT platelets, suggesting that the difference in fibrinogen binding between the thrombin-stimulated WT and PP1cγ ^−/−^ platelets were partially abolished by GSK3β inhibitor. Thus, these studies imply that GSK3 activity, in part, contributes to the decreased fibrinogen binding in PP1cγ ^−/−^ platelets.

## Discussion

Compared with Ser/Thr kinases, a specific role for PP1cγ in platelet activation is unclear. Although a compensatory up-regulation of PP1cα and PP1cβ isoforms was not particularly evident in PP1cγ^−/−^ platelets, a potential redundancy between the individual PP1c isoforms in PP1cγ^−/−^ platelet function is to be expected and may underlie the mild platelet phenotype seen in PP1cγ^−/−^ mice. Despite this constrain, our studies uncovers two novel aspects of PP1cγ with regards to the platelet biology. 1) PP1cγ regulates low dose thrombin-induced integrin α_IIb_β_3_ activation, soluble fibrinogen binding, platelet aggregation and *in vivo* thrombus formation. 2) PP1cγ is needed for a sustained thrombin-stimulated GSK3β-Ser9 phosphorylation in platelets.

Targeted disruption of the *Ppp1c* gene in mice led to impaired spermiogenesis and male infertility [19]. Although PP1cγ is more intensely studied in the area of reproductive biology, our studies indicate that PP1cγ can also regulate platelet activation in an agonist-specific manner. In particular, a subtle role for PP1cγ was noted in α_IIb_β_3_ activation, fibrinogen binding and aggregation response to low dose thrombin but not to ADP, collagen and CRP (Figures 2). Perhaps, activation of multiple redundant signaling pathways by higher concentrations of thrombin and/or other agonists might favor activation of PP1cα or PP1cβ in the absence of PP1cγ to sustain activation of platelets. More importantly, thrombus formation induced by light-dye injury was significantly delayed in the cremasteric venules of the PP1cγ^−/−^ mice (Figure 5A). Since PP1cγ^−/−^ is not a platelet specific knockout, contribution of PP1cγ from other cell types within the vasculature towards *in vivo* thrombosis cannot be ruled out. Nevertheless, this *in vivo* study is consistent and correlates well with the *in vitro* platelet data. In contrast to the thrombosis model, normal hemostasis was not altered in PP1cγ ^−/−^ mice as revealed by normal tail bleeding times (Figure 5B). It is not known whether the differential role played by PP1cγ in hemostasis versus thrombosis models was due to the intrinsic differences in the type and extent of injury in the two models, or due to the compensatory mechanisms, which was sufficient to drive hemostasis but not thrombosis.

Previous studies have identified that outside-in integrin α_IIb_β_3_ signaling mechanisms facilitated the dissociation of α_IIb_β_3_ bound PP1c, with the ability to dephosphorylate platelet myosin light chain (MLC) [10]. Following the engagement of integrin α_IIb_β_3_, PP1cγ was shown to be involved in the dephosphorylation of ADP-induced nPKCη activation [27]. These observations imply that PP1cγ might regulate platelet functions mediated by outside-in integrin α_IIb_β_3_ signaling processes. However, outside-in integrin α_IIb_β_3_ signaling dependent functions like adhesion and spreading (not shown) to immobilized fibrinogen and clot retraction were not altered in PP1cγ^−/−^ mice. Since all PP1c isoforms can interact with α_IIb_β_3_ (unpublished observation), one possibility is that the PP1cγ isoform does not participate in the functional responses initiated by outside-in integrin α_IIb_β_3_ signaling. Indeed, platelet MLC is dephosphorylated by PP1cβ/δ (myosin phosphatase) but not PP1cγ [28]. Another possibility relates to an easy compensation by other PP1c isoforms following engagement of integrin α_IIb_β_3_ to alter platelet outside-in signaling functions. Finally, the platelet functional data from PP1cγ^−/−^ mice was not completely consistent with the studies in human platelets treated with Ser/Thr protein phosphatase inhibitors. The latter studies employed calyculin A and okadaic acid and observed inhibition of platelet aggregation and secretion to various agonists and adhesion to immobilized fibrinogen [12]–[15]. These differences may stem from the fact that the platelet phenotype obtained in response to the pharmacological agents might be due to inhibition of multiple phosphatases as opposed to inhibition of only PP1cγ in this study.

Decreased platelet responses to thrombin observed in the PP1cγ^−/−^ mice was not due to altered expression of PAR3, PAR4, α_IIb_β_3_ or decreased platelet counts. In fact, we noticed a moderate but significant increase in platelet numbers in PP1cγ^−/−^ mice [Mean±SEM 596.78×10^3^/mm^3^±17.94 in WT compared with 693.71×10^3^/mm^3^±19.87 in PP1cγ^−/−^ mice; N = 12, P = 0.0003]. However, we cannot rule out the possibility that the lack of PP1cγ isoform could alter PAR4 activation associated proximal signaling events such as binding of GTP to the G proteins that couple to PAR4. Perhaps, multiple substrates of PP1cγ may exist in the signaling pathways that control α_IIb_β_3_ activation downstream of thrombin signaling. Absence of PP1cγ may alter the phosphorylation of one or more of these cellular proteins, thereby suppressing the platelet function. In fact, we noticed that the GSK3β-Ser9 phosphorylation was significantly reduced in thrombin stimulated PP1cγ^−/−^ platelets (Figures 6A and 6C). GSK3β is Ser/Thr kinase and phosphorylation of Ser9 residue inhibits its constitutively active kinase activity [29]. Although an initial study using a more generic and non selective GSK3 inhibitor implied a positive role for GSK3β in platelet function [30], a more recent comprehensive genetic analysis using GSK3β heterozygote mice revealed that GSK3β is a negative regulator of platelet function [9]. Results from the latter study indicated that thrombin stimulated GSK3β Ser 9 phosphorylation and removed the suppressive role of GSK3β on platelet function. Thus, a decreased GSK3β-Ser9 phosphorylation upon thrombin stimulation is predicted to maintain a constitutive GSK3β activity, thereby inhibiting the platelet function. Indeed, our studies revealed that the PP1cγ^−/−^ platelets stimulated with thrombin had decreased GSK3β-Ser9 phosphorylation along with decreased α_IIb_β_3_ activation, fibrinogen binding, aggregation and delayed thrombus formation. Moreover, pharmacological inhibition of GSK3β partially abolished the difference in fibrinogen binding between WT and PP1cγ^−/−^ platelets (Figure 6D). These observations suggest that additional mechanisms that underpin the decreased fibrinogen binding in thrombin-stimulated PP1cγ^−/−^ platelets exist and remain to be identified.

The molecular basis for the relationship between the loss of PP1cγ and decreased GSK3β-Ser9 phosphorylation is currently unclear. PP1cγ does not appear to directly dephosphorylate Ser9 on GSK3β, because GSK3β-Ser9 phosphorylation under basal conditions was not significantly increased in PP1cγ^−/−^ platelets. Although AKT has a major role in GSK3β Ser9 phosphorylation, PP1cγ does not appear to regulate GSK3β phosphorylation via AKT, since we observed comparable level of AKT-Ser473 phosphorylation in thrombin stimulated WT and PP1cγ^−/−^ platelets. A potential for AKT independent regulation of GSK3β-Ser9 phosphorylation was also noted in the previous study [9]. Since PKC, PKA or p90Rsk can also phosphorylate GSK3β at Ser(9) [31], it is likely that PP1cγ could affect GSK3β Ser9 phosphorylation by regulating the activation of one or more of these Ser/Thr kinases. Alternatively, PP1cγ may inhibit other Ser/Thr phosphatases such as PP2A [32], which may participate in dephosphorylating Ser9 on GSK3β under conditions of thrombin stimulation thereby ensuring increased GSK3β-Ser9 phosphorylation.

In summary, our studies indicate that under conditions of low thrombin stimulation, platelet PP1cγ is required for GSK3β-Ser9 phosphorylation, α_IIb_β_3_ activation, soluble fibrinogen binding, platelet aggregation and stabilization of thrombus formation. The agonist specific role for PP1cγ is intriguing and future studies will be needed to address how PP1cγ couples specifically to signaling by PAR receptors and not to other G-protein coupled receptors.
